# Molecular Biology of Osteosarcoma

**DOI:** 10.3390/cancers12082130

**Published:** 2020-07-31

**Authors:** Anna M. Czarnecka, Kamil Synoradzki, Wiktoria Firlej, Ewa Bartnik, Pawel Sobczuk, Michal Fiedorowicz, Pawel Grieb, Piotr Rutkowski

**Affiliations:** 1 Department of Experimental Pharmacology, Mossakowski Medical Research Centre, Polish Academy of Sciences, 02-106 Warsaw, Poland; anna.czarnecka@gmail.com (A.M.C.); ksynoradzki@imdik.pan.pl (K.S.); pgrieb@imdik.pan.pl (P.G.); 2 Department of Soft Tissue/Bone Sarcoma and Melanoma, Maria Sklodowska-Curie Institute—Oncology Centre, 02-781 Warsaw, Poland; wiktoria.firlej@gmail.com (W.F.); pawel.sobczuk@interia.pl (P.S.); piotr.rutkowski@coi.pl (P.R.); 3 Faculty of Medicine, Medical University of Warsaw, 02-091 Warsaw, Poland; 4 Institute of Genetics and Biotechnology, Faculty of Biology, University of Warsaw, 02-106 Warsaw, Poland; ewambartnik@gmail.com; 5 Institute of Biochemistry and Biophysics, Polish Academy of Sciences, 02-106 Warsaw, Poland; 6 Department of Experimental and Clinical Physiology, Laboratory of Centre for Preclinical Research, Medical University of Warsaw, 02-097 Warsaw, Poland; 7 Small Animal Magnetic Resonance Imaging Laboratory, Mossakowski Medical Research Centre, Polish Academy of Sciences, 02-106 Warsaw, Poland; 8 Interinstitute Laboratory of New Diagnostic Applications of MRI, Nalecz Institute of Biocybernetics and Biomedical Engineering, Polish Academy of Sciences, 02-109 Warsaw, Poland

**Keywords:** osteosarcoma, molecular mechanisms, targeted therapy, theranostics, tumor initiating cells, molecular imaging

## Abstract

Osteosarcoma (OS) is the most frequent primary bone cancer in children and adolescents and the third most frequent in adults. Many inherited germline mutations are responsible for syndromes that predispose to osteosarcomas including Li Fraumeni syndrome, retinoblastoma syndrome, Werner syndrome, Bloom syndrome or Diamond–Blackfan anemia. *TP53* is the most frequently altered gene in osteosarcoma. Among other genes mutated in more than 10% of OS cases, c-Myc plays a role in OS development and promotes cell invasion by activating MEK–ERK pathways. Several genomic studies showed frequent alterations in the *RB* gene in pediatric OS patients. Osteosarcoma driver mutations have been reported in *NOTCH1*, *FOS*, *NF2*, *WIF1*, *BRCA2*, *APC*, *PTCH1* and *PRKAR1A* genes. Some miRNAs such as miR-21, -34a, -143, -148a, -195a, -199a-3p and -382 regulate the pathogenic activity of MAPK and PI3K/Akt-signaling pathways in osteosarcoma. CD133+ osteosarcoma cells have been shown to exhibit stem-like gene expression and can be tumor-initiating cells and play a role in metastasis and development of drug resistance. Although currently osteosarcoma treatment is based on adriamycin chemoregimens and surgery, there are several potential targeted therapies in development. First of all, activity and safety of cabozantinib in osteosarcoma were studied, as well as sorafenib and pazopanib. Finally, novel bifunctional molecules, of potential imaging and osteosarcoma targeting applications may be used in the future.

## 1. Introduction

Primary bone cancers are a group of rare neoplasms responsible for 3–5% of pediatric cancers and nearly 0.2% of all malignant neoplasms [1]. According to SEER (Surveillance, Epidemiology and End Results–US National Cancer Institute) statistics, age-adjusted rates for new incidents have been rising by 0.4% per year in the last decade. Osteosarcoma (OS) is the most frequent primary bone cancer in children and adolescents and the third most frequent in adults, following chondrosarcoma and chordoma. The overall incidence is 3.4 per million per year worldwide [2]. An average of 900 new OS cases are reported annually in the U.S. OS affects mostly children and adolescents between 10 and 30 years of age. More specifically, OS is characterized by bimodal age distribution with the first peak at 15–19 years of age (8 cases/million/year) and the second at 75–79 years (6 cases/million/year) [3,4]. The first peak of OS in the adolescent group is due to intense linear bone growth. Tumors frequently localize in long bones, especially in areas with the most rapid growth (arms and legs, knees and shoulders). Inherited cancer predispositions syndromes also may influence high appearance of this kind of tumors in young patients [3,5]. Disruptions in signaling pathways of TP53, Rb, RecQ Like helicase 4, Bloom Syndrome RecQ Like helicase and Werner Syndrome RecQ helicase are risk factors in the pathogenesis of OS. Therefore children and adolescents suffering from genetic syndromes such as Li–Fraumeni, hereditary retinoblastoma, Rothmund–Thomson, Bloom or Werner syndrome are more prone to developing OS [4,6]. The second peak of osteosarcoma in older patients is related to higher risk of Paget’s disease of bone (PDB) and increased bone resorption by osteoclasts. In addition, history of radiation exposure (e.g., previous episodes of radiation therapy) or environmental exposures during lifetime to chemicals like radium, beryllium, chromium contribute to OS incidence in older adults [3,7,8,9,10,11].

The incidence of OS is characterized by irregular geographic pattern. Low OS prevalence is noted in selected Latin American populations as well as Asian (Indian, Japanese, Chinese) populations [12]. OS is more common in males than females in most countries with male to female ratio of 1.28:1 in the group aged 25–59 and even higher (1.43:1) in the group aged 0–24. This ratio is also varied in different populations, e.g., in Australia and Canada the incidence is even higher in males aged 75+ and in Western Europe, the OS incidence is higher in females aged 60+ than in males of the same age [2].

OS is a primary malignant neoplasm of the skeleton, affecting mostly the long bones, where sarcoma cells form immature bone or osteoid tissue. It can be divided into subtypes depending on the tumor’s features and predominant stromal differentiation (osteoblastic, fibroblastic, chondroblastic, small-cell, telangiectatic high-grade surface and extraskeletal). Depending on the histological appearance, three categories can be distinguished—high-grade, which includes most the subtypes, intermediate-grade and low-grade, including periosteal and parosteal [13]. Conventional OS is a term referring to the high-grade tumor growing intramedullary—the most frequent type, accounting for 85% of all OS cases in childhood and adolescence [4].

OS treatment requires a multidisciplinary approach and since the introduction of multimodal chemotherapy to the treatment regimen, the probability of disease-free survival for high-grade OS increased from 10–20% for surgery as the only treatment to more than 60% for multimodal chemotherapy (Chth). The current curative radical treatment combines surgery with multimodal preoperative and postoperative Chth using three or four cytotoxic agents (cisplatin, doxorubicin, high-dose methotrexate/ifosfamide) [11]. However, despite numerous attempts with different chemotherapy regimens for the past 20 years, survival rates remain essentially unchanged and no successful targeted therapies have been developed for osteosarcoma so far [11,14,15]. The 5-year survival rate in patients with localized OS reaches 70–75%, but for metastatic disease, the long-term survival rate drops to only 30% and multidrug resistance is a common problem. In the case of isolated lung metastases, surgery is the primary treatment and it must involve or at least attempt complete resection of all metastases [11,16].

In the light of developing targeted therapies and increasing the survival rate of other cancers, OS still stands where it was decades ago. Recent interest in understanding the pathophysiology and genetics of osteosarcoma resulted in several genomic studies using whole-genome sequencing (WGS) and/or whole-exome sequencing (WES). The genome analysis led to the identification of genetic heterogeneity, numerous chromosomal abnormalities, mutations and the most down- and upregulated genes, including candidate driver genes [17,18,19]. As a neoplasm with numerous chromosomal abnormalities and mutations, OS appears to be a tumor that could potentially respond to immunotherapy.

## 2. Molecular Abnormalities in Pediatric Osteosarcoma

A number of inherited germline mutations are responsible for syndromes that predispose to some neoplasms, including osteosarcomas. There are eight currently known syndromes in which osteosarcoma occurs at an increased frequency—Li–Fraumeni, retinoblastoma, Rothmund–Thomson, RAPADILINO, Werner, Bloom and Diamond–Blackfan anemia [20].

### 2.1. Hereditary Syndromes

#### 2.1.1. Osteosarcoma in Li–Fraumeni syndrome—TP53

Li–Fraumeni syndrome (LFS) was described by Frederick Li and Joseph F. Fraumeni Jr. in 1969. The cases involved children that developed rhabdomyosarcoma and had elevated frequency of various types of cancers and leukemias in first- and second-degree relatives [21]. Germline mutations in the *TP53* gene are inherited in an autosomal dominant fashion and responsible for about 70% of the cases of this syndrome. LFS is associated with soft tissue sarcomas, premenopausal breast cancer, brain tumors and many other cancers [22,23]. In Li–Fraumeni families without the *TP53* mutation, the symptoms are similar. There are a few clinical classification schemes: Classic LFS [24], Li–Fraumeni-like syndrome (LFL) [25] and criteria developed by Chompret [26]. Osteosarcomas occur at an earlier age than in the general population and develop in 5–12% of patients with LFS [24,27,28,29]. In a study including 525 families according to various criteria, families with a mutation in the *TP53* gene constitute from 14% to 56%. In the group of patients with a germline mutation in *TP53*, bone tumors were found in 0.7% [30]. Thirty-two variants of the TP53 gene were found in NGS sequencing of tumor samples from 765 patients with LFS or like-LFS [29]. Additional rare exonic variants [29] and rearrangements in the intron 1 *TP53* gene may were also reported [31]. Some Li–Fraumeni syndrome OS cases as well as sporadic OS cases were also shown to harbor heterozygous germline mutations in the *CHK2* gene [32,33,34]. Generally, there is no specific geographic pattern of Li–Fraumeni syndrome incidence. One exception is R337H mutation in the *TP53* gene that is more common in LFS/LFL families from Southeastern Brazil [35,36].

#### 2.1.2. Retinoblastoma Syndrome

The primary symptom of germline mutations (autosomal dominant) in the *RB1* gene is childhood retinoblastomas; however, in later life there is an increased risk of various neoplasms, especially OS. There are over 180 mutations causing retinoblastoma. The frequency of retinoblastoma is 1 in 18,000 live births [28]. The exact frequency of OS was initially difficult to estimate, as X-rays used for retinoblastoma treatment greatly increased the OS risk. However, even without X-rays OS are considerably more common than in the general population; the age of occurrence is similar to that in sporadic cases [20]. Specifically, the incidence of OS in hereditary retinoblastoma patients 400-fold higher than in the general population [37]. Somatic *RB1* mutations are also frequently occurring in OS patients, in a range of between 30% to 75% [38]. Recently, a few osteosarcoma cases have been described by Imbert-Bouteille et al. [39] where a low penetrance germline mutation in the *RB* gene caused osteosarcomas as the first detected tumor, without the previous occurrence of retinoblastoma.

#### 2.1.3. RECQ Disorders

Syndromes with an increased osteosarcoma risk are caused by germline mutations in genes encoding DNA helicases of the RecQ family. These germline mutations are recessive, and the syndromes they cause are very rare. These are Rothmund–Thomson type II, RAPALIDINO, Werner and Bloom syndromes (Table 1).

**Rothmund–Thomson type II (RTS II) and RAPALIDINO syndromes.** These two syndromes are both caused by mutations in the *RECQL4* gene—that possess single-stranded DNA annealing activity and functions in DNA repair. Analysis of 33 RTS cases revealed an association between *RECQL4* gene truncation (but not nonsense or missense point mutations) and osteosarcoma development [40].

RTS II patients have among others poikiloderma, sparse hair, short stature and cataracts. About 30% of them develop osteosarcomas at age from less than 10 years to 14 years [20,41]. The first review reported 61 cases of cancer among all RTS patients, of which 38 (62%) were osteosarcomas. Out of 38 cases, 3 were multicentric (metachronous) osteosarcomas; 12—developed before the age of 10, while the overall median age at presentation was 14 years [42].

RAPALIDINO syndrome (radial aplasia or hypoplasia; patellae aplasia or hypoplasia and cleft or high archer palate; diarrhea and dislocated joints; little size and limb malformations; long slender nose and normal intelligence) is very rare. The disease is found mainly in Finland, and there are very few cases, but 2/15 cases developed osteosarcoma [43].

**Werner syndrome.** This disease, which is a progeria syndrome, is caused by mutations in the *WRN* gene, encoding a RECQ helicase. In contrast to most other osteosarcoma presentations in syndromes, the tumors develop not in children but in patients between the ages of 35 and 57 years. The prevalence of this disease is much higher in Japan (1 in 20,000 to 1 in 40,000) than in the rest of the world (about 1 in 200,000). Osteosarcomas are not really frequent, they constituted 7% of tumors in Werner syndrome patients, and the others were soft tissue sarcomas, meningiomas, thyroid cancers, melanomas and myeloid disorders [20]. Japanese WS patients develop osteosarcomas at an older age than in other WS populations—between 35 to 57 years, mostly in the foot and ankle and additionally in head and neck region (i.e., patella), but not so often in long bones—a typical location for osteosarcoma [44].

**Bloom syndrome.** This syndrome, first described in 1995 [45], is related to mutations in another DNA helicase gene, *BLM* (*RECQL3*). It mainly causes carcinomas, leukemias and lymphomas, but the frequency of osteosarcoma is much higher than in the general population [20]. Bloom syndrome prevalence rate in Ashkenazi Jews is much higher than in other populations, i.e., 1% [46].

#### 2.1.4. Diamond–Blackfan Anemia

**Diamond–Blackfan anemia.** This syndrome, in contrast to the previous ones, is due not to a mutation in a tumor suppressor gene or a DNA helicase but is classified as a ribosomopathy—a disease due to a mutation in a gene encoding a ribosomal protein. The condition has varied presentation and only for 50% of cases, the genetic causes are known. The first one to be identified was a mutation in the gene encoding ribosomal protein S19; subsequently mutations in eight other genes encoding ribosomal proteins were discovered. Thirty-three osteosarcomas have been described in 608 cases, which indicates that the syndrome increases osteosarcoma risk [47]. In another study, six cases of OS have been reported among 700 Diamond–Blackfan anemia patients [48].

### 2.2. The Most Frequently Mutated Genes in Pediatric Osteosarcoma

#### 2.2.1. TP53

*TP53* is the most frequently altered gene in cancers, also in sporadic OS cases. Mutations in this gene were observed in approximately half of all tumors [50,51]. The p53 protein (encoded by *TP53* gene) regulates the cycle of cell growth, DNA replication and cell division. When DNA is damaged by ionizing radiation, activation of p53 occurs. Growth signals from oncogenes activate p53. Chemotherapeutic drugs, UV light or protein–kinase inhibitors drive to inhibition of its degradation and increase of its concentration in a cell. p53 protein binds to DNA sequences and activates expression of particular genes. They are involved in cellular processes, like cell-cycle inhibition, apoptosis, genetic stability and vessel formation. These crucial processes are unsettled in cancer cells, and they may result in inactivation of p53 directly by mutation or by inactivation of proteins that interact with p53. Moreover, posttranslational modifications have an impact on structure, stability and function of p53. Inhibiting this regulation by mutations may also drive to tumor development [52].

**Figure 1:**
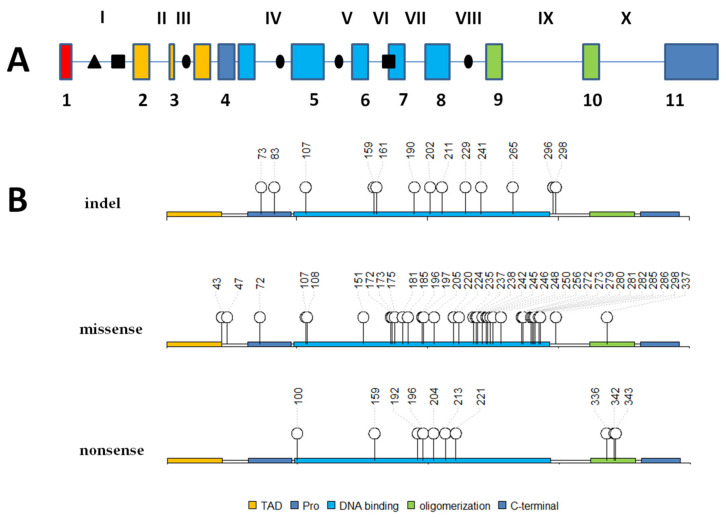
Schematic representation of the genomic locus of the *TP53* gene and its protein sequence with marked gene alterations present in osteosarcoma patients derived samples. (Panel **A**) represents mutations (●), deletions (■) and translocations (∆) locations in particular introns. Roman numerals refer to introns, Arabic numerals refer to exons. (Panel **B**) represents p53 protein primary structure with marked domains and amino acids number of mutations (indels, missense, nonsense). TAD—transactivation domain; Pro—proline-rich region; DNA binding—core domain which can bind DNA; oligomerization—oligomerization domain. Based on data from [17,18,29,62,63].

In several cancers, most mutations in the *TP53* gene are missense mutations and are localized in the DNA binding domain. In osteosarcoma, mutations of *TP53* were found with a frequency of about 20% [53]. Figure 1 illustrates the *TP53* gene locus with marked alterations found in osteosarcoma patients derived samples (also from LFS and LFL patients). Mutations in p53 may result in loss of it function or gain-of-function-mutated p53. An example gain-of-function mutation is R175H (dominant negative) that is generally common in broad spectrum of cancers. It was also present in osteosarcoma patients derived samples. In vitro studies suggest that this mutation promotes centrosome amplification connected with disturbing mitosis, cytokinesis and increase of chromosome segregation errors (aneuploidy). Expression of p53 R175H mutant results in reduced occurrence of apoptosis [54,55] Expression of other p53 mutant R273H found in osteosarcoma patient derived samples increases the frequency of gene amplification, probably by the involvement of mutated protein with topoisomerase I interaction. Gene amplification and genomic rearrangements would lead to the development of drug resistance [56]. Increase of cisplatin resistance at low concentrations (2.5 µg/mL) was observed in vitro for both these mutations. Expression of mutated R175H p53 resulted in increased resistance to etoposide other than expression of R273H p53. [57]

Alterations such as structural variation (SV) and somatic nucleotide variants (SNVs) were found in 74% of osteosarcomas [58]. In the *TP53* gene, structural variations are frequent in pediatric osteosarcoma. Structural variations (SVs) were mostly translocations with breakpoints within the first intron (Figure 1) in which inversion of a 445 kb fragment was found. These alterations were observed mainly in osteosarcoma and resulted in the loss of expression of the *TP53* gene [18,59,60,61].

Mutations in introns (III,IV,V,VIII) were characterized as splice site changes. Deletion of 31 bp fragment was observed in intron VI to exon 7 and resulted a frameshift effect [62].

There were several interesting rodent animal models with a mutation in TP53 that develop osteosarcoma. In a mouse model with mutation p.R172H (p.R175H in humans) spectrum of tumors that were developed are predominantly osteosarcomas [64,65]. The rat model named 344-Tp53tm1(EGFP-Pac)Qly/Rrrc (Fisher background) develops osteosarcomas with a frequency of 57% and 36% in homozygous and heterozygous animals, respectively. In this model, tumors are highly representative of human disease (radiographically and histologically, long bones tumor localization, frequent pulmonary metastases) [50]. In another rat model Tp53Δ11/Δ11 (Sprague Dawley background) the tumor spectrum includes multiple sarcoma types, among others osteosarcoma [66]. Heterozygous rats Tp53C273X/+ (Wistar background) predominantly develop osteosarcomas [67]. The tumors developed in this model were characterized by elevated 18F–FDG (fluorodeoxyglucose) uptake in comparison to adjacent tissues, so they are clearly visible in positron emission tomography scans [68].

#### 2.2.2. RB

Several genomic studies showed frequent alterations in the *RB* gene in pediatric OS patients, mostly regarding structural variations and fewer point mutations [18]. *RB* mutations occur in 70% of sporadic OS cases. The most common alteration is loss of heterozygosity (LOH) at 13q presenting in 60–70% of all cases [69]. LOH in the *RB* gene is considered a poor prognostic factor in OS patients [70]. Other *RB* alterations include structural rearrangements presenting in 30% of all the cases and point mutations observed in only 10% of cases [71]. Other components of the RB-pathway—including INK4, CDK4, p16 and cyclin D1—may also be subject to genetic alterations [69]. Approximately 10% to 15% osteosarcomas present *INK4* gene deletions and loss of p16 expression [72]. Walkley et al. [73] showed that the targeted p53 and Rb mutation in murine osteoblasts is sufficient to induce metastatic osteosarcoma with features of the human disease. Mutations in the *TP53* gene of these cells play a decisive role in proliferation, compromising the maturation, negatively regulating their differentiation and interfering with cellular processes such as ontogenesis [74].

#### 2.2.3. CDK

During sarcoma genesis, the loss of CDK inhibitors is a common event. Frequent alterations in CDK-related tumor suppressor genes (*TP53, RB1, CDKN2A, PTEN*) are deletion, loss of heterozygosity and mutations. Amplifications of related oncogenes (*CDK4, MDM2, MYC, TWIST1, CCND3, CCNE1*) occur as well [75]. Amplification of 12q13-15 region containing *CDK4*, *MDM2*, *SAS* and other potential oncogenes is present in 10% of osteosarcomas and is associated with ring chromosomes in parosteal OS [69]. *CDK4* is highly expressed in OS and its elevated expression correlates with the metastatic potential and is associated with poor outcome, it is considered to be a factor correlating with chemotherapy response. Additionally, *CDK4* expression makes tumors more prone to targeted therapy. Inhibition of CDK4 decreases cell proliferation and migration along with arresting the cell cycle and inducing apoptosis in human OS cell lines [76].

#### 2.2.4. c-Myc

More than 10% of OS cases possess mutation in the c-Myc gene (avian myelocytomatosis viral oncogene homolog). MYC family genes amplifications are more common in PDS-related OS than primary osteosarcoma [77]. c-Myc play a role in OS development and promote cell invasion by activating MEK–ERK pathways. It is significantly more upregulated in metastatic samples in comparison to nonmetastatic samples, indicating its major role in metastasis and therefore association with poor prognosis. In OS cells high expression of MYC promote cell proliferation, migration, clonogenicity and spheroid formation [78,79].

Myc expression in OS tumors correlates with the formation of metastasis and poor prognosis. Using osteosarcoma cell lines U2OS, 143B Chen et al. [79] showed that targeting MYC-driven super enhancers (CDK7 with THZ1 and BET family with JQ1) can successfully inhibit proliferation, migration and invasion of OS cells. In a nude mouse xenograft model with 143B cells, THZ1 caused significant suppression of tumor growth along with a drastic decrease of Ki67 cell proliferation marker.

#### 2.2.5. TGFB

It is commonly accepted that the role of transforming growth factor-β (TGF-β, encoded by *TGFB* gene) in the pathogenesis of carcinomas depends on the stage of the disease. In premalignant tumors, it can act as a tumor suppressor by increasing the expression of cyclin-dependent kinase inhibitors (mainly p21^Cip1^ and p15^lnk4b^) and/or reducing expression of regulator gene *c-Myc* and proliferative factor cyclin-D. On the other hand, in later stages of the disease, TGF-β plays the role of a tumor promoter, being able to stimulate growth and metastatic progression [80,81,82]. However, in the case of mesenchymal cell proliferation, especially in OS, this duality of function is not observed and TGF-β seems to only have a protooncogenic effect [83]. Osteolytic factors produced by osteosarcoma cells, such as interleukin-6 (IL-6), IL-11, receptor activator of NF-κB ligand (RANKL) and tumor necrosis factor-α lead to bone degradation and in consequence release of TGF-β into the tumor microenvironment [80].

## 3. Molecular Abnormalities in Adult Osteosarcoma

The spectrum of changes that occur in the genetic landscape of adult osteosarcoma is very broad. Osteosarcoma cells carry numerous loss of heterozygosity (LOH), which results in variable gene copy numbers. This type of cancer is distinguished by the amount of gene amplification. It may be due to the fact that these are amplifications generated by chromothripsis. Another phenomenon observed in osteosarcoma, called kataegis, is connected with the arisal of hypermutated regions.

### 3.1. Gene Mutations and Potential Biomarkers

Circulating DNA released from cancer cells carries much important information about changes in its structure such as deletion, insertion or translocation. This information can be easily detected in the blood of patients with osteosarcoma. Attempts to collect such a fluid biopsy and its evaluation were performed for the assessment of mutations in the *TP53* gene [84].

Recently, much data have been obtained from whole genome sequencing, whole exome sequencing and whole-transcriptome sequencing for OS samples [17,18,58,85]. These studies mostly concern pediatric cases or do not distinguish between pediatric and adult cases. It has not been fully determined whether the alterations contributing to the development of osteosarcoma in children and adults are similar [4,86]. By introducing such a division, we note that in children’s samples, most tumors have a mutation in the *TP53* or *RB* gene. Based on the data from the genome analysis, we can distinguish other genes in which changes were observed and which are listed in Table 2.

In some tumors (approximately 14%) of adult patients, IGF1 receptor amplification was observed [86]. The study on bone samples derived from osteosarcoma patients (tumor and normal) and FFPE (formalin fixed paraffin-embedded) biopsy material was performed without division into age groups. The results revealed 3300 overexpressed genes and nearly 2000 with reduced expression in osteosarcoma. The highest difference in expression between tumor and normal tissue referred to genes: *BTNL9*, *MMP14*, *ABCA10*, *ACACB*, *COL11A1* and *PKM2*, but the study was performed on samples derived from 18 patients, so the obtained results should be verified in a larger cohort [19].

Attempts are made with the help of functional tests to determine the occurrence of mutations and the order that is necessary for the initiation and development of the disease. Genes whose alteration may lead to cancerous process are summarized in Table 3. A mutation in some inductor genes could promote tumorigenesis and proliferation of altered cells. These inductor genes are called “driver” genes. The group of these osteosarcoma inducers (the first driver) includes *TP53, NOTCH1, MYC, FOS, NF2, WIF1, BRCA2, APC, PTCH1* and *PRKAR1A*. However, the potential to induce osteosarcoma in each of the above genes is different. Animal experiments showed that mutations in a pair of genes: *WIF1* and *BRCA2* resulted in a negligible effect of tumor formation, in contrast to a mutation in a pair of *TP53* and *NOTCH* genes [73,88,89,90].

Synergistic genes (also called synergistic drivers) are those whose independent dysfunction cannot start the cancerous process. Tumor initiation and growth may occur when a synergistic driver coexists with a primary driver, also a synergistic driver could be a germline mutation, then the first driver could be damaged by a somatic mutation. The group of synergistic genes in OS includes *RB1*, *TWIST*, *PTEN* and *JUN* [91].

Another group that could be potential biomarkers of osteosarcoma are alterations in mRNA or protein expression levels. The candidate biomarker may be cathepsin D. Overexpression of this protein was observed in osteosarcoma tumor samples and samples from lung metastases [92]. Low expression level in osteosarcoma is found for FBXW7 mRNA and protein. In vitro and in vivo studies showed that the increased expression of FBXW7 is associated with a decrease in the proliferation rate of tumor cells and a slowdown in tumor growth [93]. In contrast, a high expression level was noticed for HMGB1 protein in tumor cells (high-mobility group (non-histone chromosomal) protein 1), for miR-421 in osteosarcoma tissues [94] or high level of Gla matrix protein in serum. These potential biomarkers correlate with poor prognosis and poorer survival rates [95,96].

### 3.2. Chromothripsis and Kataegis

**Figure 2:**
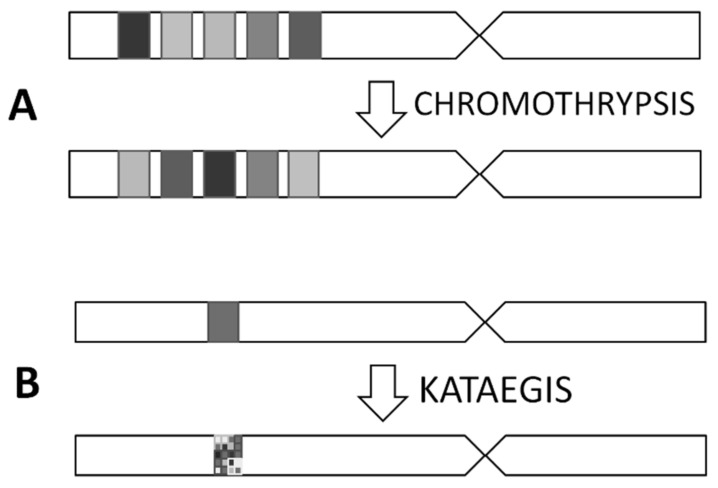
Schematic representation of changes after chromothripsis (Panel **A**) and kataegis (Panel **B**). The occurrence of chromothripsis results in a new configuration in the part of a chromosome. Kataegis describes a hypermutation pattern located in one or multiple loci in the genome.

Chromothripsis is a type of genetic abnormality which consists of fragmentation of the chromosomal region and setting it in a new configuration (see Figure 2). It results in several genomic rearrangements in one or more chromosomes. The frequency of chromothripsis in osteosarcoma is high, about 77% [97]. This abnormality in osteosarcoma generates amplification (*CDK4*, *MDM2*, *COPS3*), gains (*RICTOR, TERT*) or disruption (*TP53, NF1*) of driver oncogenes and is localized in chromosomes 5,6,12,13,14,17 [18,86].

The kataegis is a result of elevated mutation prevalence over regions in chromosomes (see Figure 2). In osteosarcoma, the prevalence of kataegis estimated on the basis of small cohort of samples was high-ranging between 50–85%. This phenomenon is common in breast cancers. Osteosarcoma patient derived samples share the same several characteristic features described in breast cancers, like the same type of substitutions, the same class of mutation, occurrence of hypermutated regions with structural variations breakpoints or rearrangement sites, occurrence of macro- and microclusters in hypermutated regions. Kataegis was not common in regions of *TP53* or *ATRX* genes, the most frequently mutated genes in OS [98,99].

## 4. Small RNAs in Osteosarcoma

Small RNAs have the potential to become prognostic biomarkers of OS (listed in Table 4) [100,101,102]. An example of this kind of RNA deregulated in osteosarcoma is miR-421 [94]. Overexpression of this molecule results in proliferation, migration and invasion of tumor cells [94,103]. The expression level of miR-421 measured in serum is higher in patients with OS than in healthy volunteers. In most OS patients high miR-421 expression occurs within the tumor [94]. Other microRNAs of potential significance in osteosarcoma development are: miRNA-129-5p (miR-129-5p), miR-330-3p, miR-365, or miR-491-3p [94]. Another molecule of potential importance is miR-191 which expression is elevated in OS. In vitro experiments demonstrated that miR-191 promotes proliferation of cells and Chk2 kinase is its direct target [104]. Other molecules include miR-21, -34a, -143, -148a, -195a, -199a-3p and -382 that regulate the activity of MAPK and PI3K/Akt signaling pathways [105]. In vitro study with SOSP-9607 and Sais-2 OS cell lines showed that overexpression of miR-34a inhibited the proliferation, migration and invasion of cells, whereas *in vivo* study using these cell lines showed a decrease in tumor growth and pulmonary metastases as a result of miR-34a overexpression [106]. Plasma levels of miR-195-5p, miR-199a-3p, miR-320a and miR-374a-5p were proven to be preoperatively increased in OS patients, whereas after the resection their levels significantly dropped [107].

In the study of two large cohorts, Andersen et al. [101] identified 24 downregulated and 5 upregulated miRNAs. Some of them have been described as oncomiRs in other types of malignancies (miR-181a-5p, miR-181c-5p, miR-223-3p and miR-342-3p).

Downregulation of miR-374b and miR-543-3p and upregulation of miR-126 is associated with OS invasion and metastases and promotes angiogenesis by increasing expression of VEGF-A and angiopoietin-2 [110,111,112,113]. Downregulation of miR-539-3p, miR-218, miR-143-3p, miR-150 and miR-183 enhance the potential of cells to invade and migrate, among others by increasing expression of the matrix metalloproteinase 8 (MMP-8), MMP-2,-9, MMP13 and ezrin [74,111,114,115]. Another miRNA enhancing the metastatic potential of OS is miR-23. Increased through TGF-β stimulation (produced in OS cells) miR-23 levels in cytotoxic T-lymphocytes cause a loss of their antitumor effect and thus decrease the immune response to circulating cancer cells [116]. When it comes to the adherence of circulating OS cells to the vascular endothelium in the final stages of metastatic development, it is suggested that decreased miR-329 expression may support cell adhesion by increasing CD146 expression [111]. Moreover, overexpression of miR-148a in OS can cause downregulation of GADD45A which is associated with multidrug resistance, whereas miR-34a can increase drug resistance by targeting c-Myc and activating PI3K–AKT and RAS/MAPK pathways [108].

It is a challenge to determine a suitable and reliable micro-RNA biomarker for osteosarcoma because it requires meta-analysis and validation in prospective studies. A marker easily detectable in the patient’s blood may be used in the future for developing diagnostic tests [117]. Besides being a potential biomarker, miRNAs profiling may provide a theoretical basis for establishing novel therapeutic targets. Differently expressed miRNAs target genes important for the OS that are involved in intracellular-signaling pathways such as the c-Met, Notch, RAS/p21, mitogen-activated protein kinase (MAPK), Wnt and Jun/Fos pathways [118]. For example, MiR-524 and miR-221 enhance the proliferation of OS cells via inhibition of the target gene *PTEN* and activation of the PI3K/AKT pathway [119].

## 5. Role of Tumor Initiating Cells in Osteosarcoma

TICs (in oncology referred to as cancer stem cells) are characterized by the potential to self-renew, high tumorigenicity in nude mice and the ability to efficiently reconstitute all tumor subpopulations as well as the primary tumor phenotype [120,121,122]. TICs are responsible not only for the development of malignancy, but also disease recurrence, progression and metastatic spread, as well as aggressiveness, resistance to chemo- and radiotherapy and targeted treatment [123,124].

**Figure 3:**
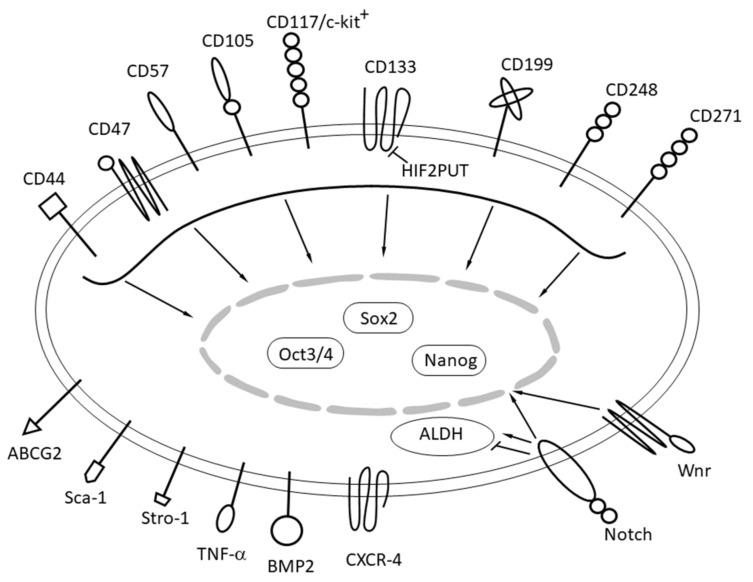
Overview of molecular markers of osteosarcoma tumor initiating cells.

TICs are capable of initiating sarcomagenesis [125,126]. The TIC subpopulations emerge after the accumulation of epigenetic and genetic alterations in a cell within the aberrant population, initially generated by the sarcoma cell-of-origin. TICs have been described in OS, but also in chondrosarcoma, Ewing’s sarcoma and synovial sarcoma [127]. These TICs are characterized by the expression of typical stem-related markers (*OCT3/4*, *NANOG* and *SOX2*; Figure 3) [128]. TICs also display tumor re-initiating properties as they self-renew and sustain tumor growth in serial transplantation experiments. In addition, TICs presence is associated with tumor drug resistance and metastatic growth, which may finally trigger the relapse of sarcomas [127]. TICs are therefore responsible not only for sarcoma development, but also disease recurrence, metastatic spread and progression. TICs may probably also be regulators of sarcoma aggressiveness due to internal resistance to chemo- and radiotherapy as well as resistance to targeted drugs and immunotherapy [129,130,131].

TICs have been primarily identified in OS over the last 10 years in cell lines, as well as in tissues obtained from tumor surgery. Although the cell biology of OS is still under investigation and needs further research, basic information has been confirmed by different sarcoma study groups. CD133+ cells have been shown to be present in two human primary cultures of OS cells and exhibit stem-like gene expression (e.g., *OCT3/4* and *NANOG*), sphere formation and side population properties [132]. CD133 may be a marker of TICs in OS. CD133+ cells have been identified in three OS cell lines (Saos2, MG63 and U2OS). CD133+ cells have been further characterized to be more proliferative, overexpress Oct3/4 and ABCG2, have a small side population fraction and form spheres in serum-free conditions [133]. Cells isolated from CD133^+^-derived spheres form large tumors *in vivo* [132]. Other markers used to isolate TICs from OS include CD117/Stro-1 and CD271. CD177+ cells were isolated from K7 M2, KHOS/NP, MNNG/HOS, 318–1, P932 and BCOS OS cell lines and present a phenotype of sphere formation, drug resistance, stem-like gene expression, in vivo tumorigenicity and metastatic potential [134]. At the same time CD271+ cells were found in human primary cell lines, as well as in the MNNG/HOS, U2OS and Saos2 cell lines. CD271+ cells present with stem-like gene expression, sphere formation, drug resistance and in vivo tumorigenicity [135]. As the CD133+ marker is the most recognized marker of OS, TICs characterized by the expression of 1GFBP3, Oct 4, alkaline phosphatase, ALDH, TERT, SSEA 3/4 and CD133 can be used.

It has been shown that TICs express mesenchymal stem cell (MSC) markers and retain pluripotent properties in vitro [136] and several methods have been developed to isolate and enrich subpopulations with tumor initiating cell properties within the tumors [137]. Sphere (sarcosphere) cultures in anchorage-independent media, that continuously self-renew and are capable of secondary sphere formation and have enhanced tumorigenicity *in vivo* were shown to be a feasible method to enrich TICs in vitro in selected sarcomas. At the same time, CD133 has been suggested as a TIC marker in multiple sarcomas (osteosarcoma, Ewing sarcoma, chondrosarcoma, synovial sarcoma). Moreover, the CD133^+^CD44^+^ cell subpopulation is even more aggressive in terms of sphere formation, migration and invasiveness as well as characterized by high tumorigenicity. High expression of these tumor initiating cell markers has also been reported in clinical samples. Other suggested TIC markers are CD248 (endosialin), CD117(c-KIT) and CXCR4 (Figure 3). Nevertheless, all these experiments have been limited in number, reported in selected sarcoma subtypes and the results are contradictory in some cases. The final surface marker defined phenotype of TICs needs to be established, in particular in relation to genetic/genomic abnormalities present in sarcoma cells. Moreover, it is also highly probable that surface biomarkers are also significant for TIC physiology and in fact by intracellular signaling promote not only a stem-like phenotype, but also drug resistance, migration or invasive potential of these cells.

Additional markers should also be present on TICs as according to the International Society for Cellular Therapy (ISCT) mesenchymal stem cells (MSCs) should be positive for CD73, CD90 and CD105, but negative for CD34, CD45, CD11b or CD14, CD19 or CD79α and HLA-DR. The transcription factors Oct-4, Sox2 and Nanog are capable of inducing the expression of each other and are essential for maintaining the self-renewing undifferentiated state of cells [131,137,138].

## 6. Role of Cell–Cell Interactions in Osteosarcoma

In OS, as in other tumors, multiple cell–cell interactions between the OS malignant cell and tumor stromal cells are important in tumor development and progression. OS tumor niche is built of OS cells, bone cells (osteocytes, osteoclasts, osteoblasts), stromal cells (fibroblast-like cells, mesenchymal stromal cells–MSCs), vascular cells (endothelial progenitors cells–EPCs, endothelial cells, pericytes), immune cells (monocytes, macrophages, lymphocytes) and extracellular matrix (ECM) with cytoskeletal proteins and calcifications. Multiple cytokines, growth factors, chemokines as well as their receptors have been reported as significant for OS cells’ proliferation. In autocrine and paracrine mode these molecules stimulate cell division and differentiation of cells including not only OS cells, but also MSCs, osteoblasts and endothelial cells [139]. In OS intratumoral interleukin 6 (IL-6), interleukin 8 (IL-8), stromal derived factor 1 SDF-1 (known also as C–X–C motif chemokine 12—CXCL12), chemokines (C–C motif) ligand 5 (CCL5) and vascular endothelial growth factor (VEGF) promote OS cell growth and angiogenesis in the tumor, as well as metastatic spread [140,141]. Most of all OS tumor microenvironment is characterized by an abundance of transforming growth factor-β1 (TGF-β1). This factor induces non-stem-like OS cells to adopt sarcoma stem cell phenotype, and stem cells promote progression and chemoresistance as described above [142]. As per bone microenvironment, key regulators of bone metabolism are osteoclasts and osteoblasts. In fact, osteoblasts in OS niche secrete multiple components of ECM and matrix metalloproteinases (MMPs) [143]. Moreover, OS is a disease with deregulated Receptor Activator of Nuclear factor κB Ligand (RANKL) and its receptors RANK and osteoprotegerin (OPG) signaling. In its canonical function RANKL, secreted by osteoblasts, induces bone destruction by mature osteoclasts. In response osteoblasts secrete OPG—RANKL decoy receptor—and in this way inhibit osteoclast differentiation and resultant bone resorption. In OS RANKL/RANK/OPG-signaling is associated with the pathogenesis of osteosarcoma directly through RANK on the osteosarcoma cell surface and indirectly by regulating osteoclast activities. RANKL/RANK-signaling regulates OS cell migration and tissue-specific metastatic behavior in the lungs, but has no direct impact on OS-associated bone destruction and does not impact OS cell proliferation [144,145,146]. The second important group cells in OS tumor niche are osteosarcoma-associated fibroblasts (OS–AF) deriving from mesenchymal stem cells (MSC). OS–AFs promote OS cells’ motility, transendothelial migration and invasiveness. Intratumoral fibroblasts activated in reciprocal cell–cell interaction by OS cells, secrete IL-6 and -8, monocyte chemoattractant protein MCP-1 (CCL2) and growth-regulated oncogene GRO-α (CXCL1) [147,148]. IL-6 secreted by OS–AF is to activate STAT3-signaling in OS cells, and by intracellular-signaling network promote cell proliferation, migration, invasion, and as a result also development of pulmonary metastases. Moreover, IL-6 paracrine-signaling increases expression of a multidrug-resistant protein (MRP) in OS cells and favor drug resistance [148,149]. Both OS cells and OS–AF cells were shown to release extracellular vesicles (EV) or exosomes from the plasma membrane. EVs secreted by OS–AF contain tumor supportive microRNAs (including miR-148a and miR-21-5p) and multiple proteins, as well as metabolites (lactate, glutamate). OS–AF derived EVs were shown to increase OS cell survival and migration, especially under stress. OS cells derived EVs promote angiogenesis, cell adhesion and migration of OS-AF cells subpopulation [147,150,151]. OS–AF are also involved in OS tumor metabolic reprogramming. These cells secrete within niche glycolytic metabolites, including lactate and ketones that are energy-rich. OS cells are uptake such metabolites and burn in the Krebs cycle in reverse Warburg effect for ATP synthesis increasing bioenergetic potential. Lactate up-take also increases the migratory potential of OS cells [152]. OS and stromal cells regulate also differentiation and migration of EPCs and promote neo-vasculogenesis, but the secretion of angiogenesis-related factors such as VEGF, TGF-β1, monocyte chemoattractant protein 1 (MCP-1), Activin A and osteopontin (OPN) [153]. In particular, autocrine VEGF–VEGFR-1-signaling was shown as associated with tumor angiogenesis, but also with increased tumor growth [154]. In turn tumor vasculature regulate immune infiltration. VEGF and angiopoietin (ANG2)-signaling promote intratumoral vascular instability that actually limits leukocyte extravasation and tumor infiltration. Therefore, T cells are largely excluded from the metastatic tumors. Such OS tumors are immunologically cold [155]. Even more immune infiltrates that were found in osteosarcoma tumors promote actually locally immune tolerant environment due to dysregulation of the M1/M2 macrophage balance with the pro-metastatic profile of CD163^+^ M2 abundance [156]. Finally, tumor immune-infiltration is regulated by ECM and MMP-dependent proteolysis described first, [143].

## 7. Molecular Signature of Osteosarcoma Metastases

OS is characterized by its early metastatic potential. At the time of diagnosis, 20% of OS patients have already developed metastases, out of which 90% are lung metastases. The 5-year survival rate of patients with distant metastases drops to only 15–30% [157]. The mechanism of OS metastasis development is complex and requires interaction between multiple genes and signaling pathways. There are few identified factors contributing to invasion and metastasis, such as deletion of the *TP53* gene and activation of the Notch pathway in OS cells. Other studied factors include the CXCR4/CXCL12 pathway, overexpression of ezrin and MET, induction of Src-family tyrosine kinase (SFK) [158,159,160,161,162,163]. All of them could be considered as potential therapeutic targets.

Many studies screen metastatic and nonmetastatic groups or cell lines for differentially expressed genes (DEGs), revealing heterogeneity between cells with high and low metastatic potential. Muff et al. [164] analyzed DEGs by microarray analyses in the four human OS cell lines SAOS/LM5, HUO9/M132, HOS/143B and MG63/M8 and the two mouse cell lines Dunn/LM8 and K12/K7 M2. In the two metastatic osteoblastic cell lines systems Dunn/LM8 and SAOS/LM5, they identified 48 genes, which can be considered to be relevant in osteoblastic OS metastasis. Seventeen of these genes were frequently upregulated in metastatic cell lines in comparison to the corresponding parental cell lines (including *SERPINE2*, *FHOD3*, *PAX3*, *DLX4*, *FOXQ1*, *LOX*, *PCBD1*, *EHF*), whereas 31 genes were downregulated (including *CCDC80*, *DAB2*, *TGFB2*, *SLC1A3*, *OSMR*, *TPM1*, *DEPDC6*, *PHLDA1*).

Results by Tian et al. [165] using weighted gene co-expression network analysis (WGCNA) suggest that insulin-like growth factor binding associated genes (including *IGFBP5*, *IGFBP6*, *WISP3*, and *MYL2*) may play important role in the OS metastatic process. In the study conducted by Li et al. [157], 24 downregulated DEGs were identified. Three of them—*ALOX5AP*, *CD74* and *FCGR2A*—were suggested to be the candidate genes with prognostic value. Interestingly, their expression was higher in the lung and lymph node tissues than, cancer tissues. This indicates that these candidate genes are probably expressed in the microenvironment of the tumor.

## 8. Molecular Imaging and Theranostic Strategies for Diagnosis and Tracking of Treatment Efficacy in Osteosarcoma

Most cases of OS are primarily diagnosed by medical imaging due to pain or as an incidental finding. The diagnosis is most often initiated by a classical X-ray check-up and later confirmed by an X-ray CT scan. CT enables an assessment of local tumor size and distant metastases, but soft tissue invasion is less clearly visualized. Therefore, MRI may provide additional information on local tissue infiltration, including locations of arteries or veins and tumor infiltration, intraosseous size of the tumor and size of the soft tissue component, neurovascular structural infiltration and allow to detect metastases. Finally, MRI is also used in patients with contraindications to I-based contrast agents. Dynamic MRI is reliable for evaluating changes in tumor vascularity and to provide additional information on the tumor response to primary chemotherapy. The clinical value of diffusion MRI is currently under evaluation [11].

Several types of new promising approaches are currently in development for imaging of osteosarcomas. First of all, various positron emission tomography (PET) tracers have been proposed in addition to a standard technique based on (18F) fluorodeoxyglucose. One is (18F) sodium fluoride that visualizes calcium metabolic activity and is currently used for detecting bone metastasis [166]. A recent clinical trial has shown a correlation between the (18F) sodium fluoride-PET results and overall survival, while in FDG–PET there was no correlation [167].

Another recent approach is based on hyperpolarized MRI tracers. Sensitivity enhancement achieved by hyperpolarization of nuclei of a metabolic tracer offers the possibility of using noninvasive magnetic resonance spectroscopy (MRS) and MRI to measure fluxes through individual enzyme-catalyzed reactions. In sarcoma animal models it was observed that quantitative reduction in lactate production after administration of hyperpolarized pyruvate as monitored by hyperpolarized MRI followed the tumor size changes and was useful for monitoring of therapy in this setting [168]. Another study showed the usefulness of this approach in canine patients with sarcomas [169].

Finally, several antibody-based molecular probes are in development, e.g., detecting CXCR4- [170] or CD105 [171]. Such probes in combination with targeted treatment, i.e., a theranostic approach could be a particularly important new direction, one such example could be RGD-Bi(2) S(3) @MSN/DOX theranostic platform [172].

## 9. Targeted Therapies for Osteosarcoma

Understanding the molecular mechanisms of osteosarcoma formation and development provides information that allows us to set new targets for the molecular therapy of this type of cancer. Proteomic analyses as well as NGS sequencing that allow the comparison of the proteome and genome in sarcoma cells with normal cells enable the initial selection of potential targets for anticancer therapy [173]. Only a few studies have reported deregulation in the proteome of osteosarcoma cells while the number of known mutations is also growing. The preliminary selection of proteins that can be targets of novel drugs is presented in Table 5. Some of the listed compounds are currently evaluated in clinical trials, these are bevacizumab, sorafenib, regorafenib, pazopanib, cabozantinib, sirolimus, everolimus and glembatumumab vedotin, others require preclinical studies to assess their potential use in the treatment of osteosarcoma [174].

The frequently altered protein p53 is a potential target in osteosarcoma therapy. Despite the many compounds tested in preclinical tests, there are no drugs that restore the function of mutated p53 [91] though a gene-altering therapy that has been successfully used for other cancer types may be feasible [175]. Disialoganglioside (GD2), a molecule on the surface of tumor cells is another potential target for the treatment of osteosarcoma as almost all OS cases express a large amount of GD2. Therapy based on chimeric anti-GD2 antibody dinutuximab improves survival outcomes in patients with neuroblastoma. Currently, there are studies using cell therapy with anti-GD2 lymphocytes or testing several anti-GD2 molecules like dinutuximab, Hu3F8 and Hu14.18K322A [174]. Osteosarcoma appears to be a sarcoma subtype potentially responding to immunotherapy. In the osteosarcoma field, several studies of immune checkpoint inhibitors are currently evaluated, among others nivolumab with ipilimumab, pembrolizumab, INF-α-2b and L-MTP-PE (liposomal muramyl tripeptide phosphatidylethanolamine) have been tested [174]. Tyrosine kinase inhibitors are a group of drugs that are widely investigated in osteosarcoma, as described below.

### 9.1. Cabozantinib in Osteosarcoma

Cabozantinib is a vascular endothelial growth factor receptor (VEGFR) tyrosine kinase inhibitor (TKI) that also possesses specific MET receptor inhibitory activity. Its antitumor activity has been shown in experiments on cell lines as well as in vitro models. Besides direct inhibition of osteosarcoma cell growth and viability due to inhibition of ERK and Akt-signaling pathways, cabozantinib can affect the tumor microenvironment by reducing the production of receptor activator of nuclear factor-kB ligand (RANKL) by osteoblasts. This leads to the amelioration of growth-stimulating interactions between osteoblasts and osteosarcoma cells [176,177].

The activity and safety of cabozantinib in osteosarcoma have been studied in a multicenter, single-arm phase II CABONE trial [178]. The study was performed in patients with advanced osteosarcoma with documented progression. Adult patients received oral cabozantinib 60 mg once daily, whereas children (<16 years) 40 mg/m^2^ once daily. The study has reached its primary endpoint with 12% of patients with an objective response at six months (all partial responses) and 33% of patients nonprogressing after six months. Overall, 17% of patients had partial responses and 62% stable disease. Median PFS was 6.7 months and median OS 10.6 months. PFS rate at four months was 71% which is much better than 30%, considered as a threshold for promising therapies in osteosarcoma worth further investigations. Interestingly, 65% of patients who underwent metabolic assessment with 18F–FDG PET–CT had a partial metabolic response after the first cycle of cabozantinib what can be considered as a potential biomarker of cabozantinib efficacy in osteosarcoma. The metabolic response was associated with better PFS—patients with response had mPFS of 7.2 months, while patients with stable or progressive metabolic disease had mPFS of 4.5 and 1.8 months, respectively. Cabozantinib was generally well-tolerated. The most common grade 3 and 4 toxicities were hypophosphatemia (7%), AST increase (7%), palmar–plantar syndrome (4%), pneumothorax (9%) and neutropenia (9%) [178]. The study has shown significant activity of cabozantinib in patients with advanced osteosarcoma, especially considering the fact that most patients had received at least one line of therapy before enrolment. Moreover, 37% of patients had PFS at least 33% longer than on previous therapy, which is widely acknowledged as a marker of meaningful clinical activity [179]. Another trial with cabozantinib in patients with osteosarcoma (NCT02867592) is currently ongoing.

### 9.2. Pazopanib in Osteosarcoma

Pazopanib is a TKI with a high affinity to VEGFR, as well as activity against PDGFR, KIT and FGFR. Based on the PALETTE trial, pazopanib has been approved for second-line treatment of nonadipocytic soft tissue sarcomas after the failure of standard chemotherapy. Comparing to placebo, it has improved mPFS from 1.6 to 4.6 months. [180] Data on pazopanib efficacy in patients with osteosarcoma are limited and based on case reports and small retrospective analyses based on the off label use of the drug. Longhi et al. [181] reported a 60% disease control rate (DCR) with 1 partial response among 15 patients with relapsed osteosarcoma treated with pazopanib. Similar outcomes were reported by Aggerholm-Pedersen et al. [182] in 19 patients with bone tumors, including eight osteosarcoma patients, treated with pazopanib. Four patients with osteosarcoma had a partial response to the treatment. The mPFS in the whole group was 5.5 months, ORR 32% and DCR 68%. Efficacy of pazopanib was also shown in some case reports of pediatric [183] and adult patients [184,185,186], with PFS of approximately six months. The available data are unclear, and results need verification in randomized studies. A trial with pazopanib in combination with topotecan in the treatment of patients with metastatic soft tissue and bone sarcomas (CT02357810) is currently ongoing. Another trial with pazopanib in osteosarcoma metastasizing to lungs (NCT01759303) has been terminated due to low accrual.

### 9.3. Sorafenib in Osteosarcoma

Sorafenib is a TKi that targets VEGFR, PDGFR, and KIT. In preclinical studies, it has shown some efficacy against osteosarcoma [187,188] and thus has been tested in clinical trials. In a Phase II trial, patients >14 years old, progressing after standard treatment were receiving 400 mg of sorafenib bidaily. The primary endpoint, a PFS rate at four months, was 46%. mPFS was four months and median OS 7 months. Objective responses (all partial responses) were observed in 9% of patients while DCR was 49% [189]. In a retrospective analysis in pediatric osteosarcoma, partial responses to sorafenib were reported in six of eight patients [190]. Median PFS was four months. There was an insignificant tendency towards better outcomes when sorafenib was used in the first recurrence.

Sorafenib has been also tested in combination with mTOR inhibitor, everolimus, in a non-randomized phase 2 trial [191]. Patients received 800 mg sorafenib plus five milligrams everolimus once per day. A total of 45% of patients enrolled for the study remained progression-free at six months. Median PFS was five months and the median OS was 11 months. ORR was 10% (all partial responses) and DCR was 63%. The study is negative because it has failed to achieve the primary endpoint of six month PFS of 50% or greater, however, results are better than for sorafenib alone and exceeded generally used benchmarks for effective therapies in sarcomas. These observations show that combined therapies with molecular targeted agents can be promising in the treatment of refractory osteosarcoma, but further studies are needed.

## 10. Conclusions

Osteosarcoma occurrence is high in adolescence and also in adults older than 65 years. Although osteosarcoma occurs mostly in patients without germline mutations, hereditary syndromes associated with mutations in the *TP53, RB, RECQ, WRN or BLM* genes are also reported among osteosarcoma patients. In fact, analysis of these genes and syndromes may help understanding the molecular basis for developing this type of sarcoma. In fact, also somatic mutations in osteosarcomas occur in *TP53, RB, CDK, TGFB* genes. Osteosarcoma cells also carry numerous loss of heterozygosity (LOH) events, which results in variable gene copy numbers in osteosarcoma cells, in particular a high number of gene amplification. These amplifications are expected to occur by chromothripsis. The introduction of NGS and proteomic techniques allowed a better understanding of molecular alteration in OS and is expected to induce the development of new therapies.

An important aspect of sarcomagenesis is the contribution of tumor initiating cells. TICs have been identified in OS in cell lines, as well as in tissues obtained from tumor surgery. These cells express various molecular markers, of which CD133 seems to be one of the most important. Other crucial membrane markers are CD44 and CXCR4. Basic information has been established by different sarcoma study groups, but the contribution of TICs to sarcomagenesis and their pathophysiology definitely needs further investigation. This should lead to TIC-targeted therapies in OS treatment.

Current osteosarcoma treatment is based on adriamycin chemoregimens and surgery. Several potential targeted therapies in OS are in development. The most advanced trials concern cabozantinib, sorafenib and pazopanib. Nevertheless, these data are only preliminary and new randomized trials are needed for registration and implementation into routine clinical practice. In fact, novel drugs against osteosarcoma are expected and highly needed. It seems that molecular abnormalities currently explored in the field could be used as potential drug targets, including the deregulation of the p53 pathway as well as stem-cell related signaling. Finally, novel bifunctional molecules, of potential imaging and osteosarcoma targeting applications may be used in the future. New molecular imaging methods that are currently developed in vitro and in animal models of osteosarcomas may contribute not only to a better understanding of the pathogenetic mechanisms of this disease, but also to the monitoring of experimental therapies. In particular, approaches that combine therapy and diagnostics (i.e., theranostics) and multimodal molecular imaging could be particularly important in the future. However, to date, only a few studies have exploited targeted imaging or the theranostic approach in OS or animal models of OS.

## Figures and Tables

**Table 1:** Hereditary syndromes resulting in osteosarcoma development.

Syndrome	% of OS	Genes Mutated	References
Li–Fraumeni syndrome	12%	*TP53*	[20,28]
Retinoblastoma syndrome	7%	*RB*	[49]
Rothmund–Thomson type II (RTS II) syndrome	32%	*RECQL4*	[28]
RAPALIDINO syndromes	13.3%	*RECQL4*	[20]
Werner syndrome		*WRN*	
Bloom syndrome		*BLM*	
Diamond–Blackfan anemia	5.4% 0.9%	*RPS19, RPL5, RPL11, RPL35A,* *RPS24, RPS17, RPS7, RPS10, RPS26*	[47] [48]

**Table 2:** Comparison of altered genes in pediatric and adolescent/adult patients in osteosarcoma based on [4,87].

Process	Pediatric	Adolescent/Adult
Control of cell cycle and apoptosis	*TP53*, *RB*1, *CDKN2A*, *CDK4*, *MDM2*, *MYC*, *CARD11*, *CTNND1*, *BLM*, *CCNE1*, *COPS3*, *PRKCA*	*TP53*,
PI3K-mTOR and RAS-signaling pathways	*EGFR*, *GNAQ*, *GNAS*, *ALK*, *PDGFRA*, *PDGFRB*, *PIK3CA*, *AKT2*, *PIK3R1*, *PTEN*, *TSC2*, *VHL, CBL*	*PIK3CA*,
Notch-signaling pathway	*NOTCH1-4*, *MAML2*, *FBXW7*, *PDPK1*, *AKT1*, *E1F4B*	*AKT1,*
DNA damage repair	*BRCA*1, *BRCA2*, *MLH1*, *BAP1*, *ATM*, *WRN*	*SETD2, FBXW7*
Chromatin modification	*ATRX*, *FANCE*, *RECQL4*, *ARID1A*, *EP300*	*H3F3A*
Regulation of transcription	*Runx1*, *GAS7*, *MLLT3*	
Angiogenesis		*TIE1* and *KD*R

**Table 3:** Driver (strong inductors) and synergistic genes (need “a cooperation” with driver genes) in which alterations may lead to osteosarcoma [89,90,92,93].

Driver Genes	Synergistic Genes
*TP53, NOTCH1, MYC, FOS, NF2, WIF1, BRCA2, APC, PTCH1*, *PRKAR1A*	*RB1*, *TWIST*, *PTEN*, *JUN*

**Table 4:** Downregulated and upregulated miRNA in osteosarcoma (OS) patients [74,101,102,108,109].

Downregulated miRNAs	Upregulated miRNAs
miR-16, miR-31, miR-100-5p, miR-221-3p, miR29b-1-5p, miR-125b-1-3p, miR-29a-5p, miR-370-3p, miR-299-5p, miR-493-5p, miR-409-3p, miR-30e-3p, miR-431-5p, miR-432-5p, miR-410-3p, miR-411-5p, miR-376c-3p, miR-125b-5p, miR-335-5p, miR-376a-3p, miR-382-5p, miR-154-5p, miR-222-3p, miR-137, miR-92b-3p, miR-433-5p, miR-127-3p, miR-143, miR-143-3p, miR-539, miR-539-3p, miR-218, miR-183, miR-3928, miR-140, miR-150, miR-449c	miR-27, miR-148a, miR-181a-5p, miR-181c-5p, miR-195, miR-223-3p, miR-342-3p, miR-378a-3p, miR-21, mirR-221, miR-106, mi-R-218, miR-126, miR-574-3p,

**Table 5:** List of protein targets and potential drugs in targeted therapies of OS, based on [173].

Protein	Potential Drug
DNMT1 (DNA (cytosine-5)-methyltransferase 1)	azacytidine (Vidaza), decitabine (Dacogen)
ERBB2 (receptor tyrosine–protein kinase erbB-2)	trastuzumab (Herceptin), lapatinib (Tycerb), afatinib (GIOTRIF/GILOTRIF), pertuzumab (PERJETA)
GSR (mitochondrial glutathione reductase	carmustine (GLIADEL^®^ WAFER)
HDAC1 (histone deacetylase 1)	vorinostat (Zolinza)
HDAC2 (histone deacetylase 2)	romidepsin (Istodax)
KIT (mast/stem cell growth factor receptor kit)	imatinib (Gleevec), sorafenib (Nexavar), sunitinib (Sutent), pazopanib (Votrient), dasatinib (Sprycel), axitinib (Inlyta), nilotinib (Tasigna)
FGFR1 (fibroblast growth factor receptor 1)	lenvatinib (Lenvima)
MET (hepatocyte growth factor receptor)	cabozantinib (COMETRIQ), crizotinib (XALKORI)
MTOR (serine/threonine protein kinase mTOR)	temsirolimus (Torisel), everolimus (Afinitor)
PARP1 (poly (ADP–ribose) polymerase 1)	olaparib (AZD2281)
PDGFR α (platelet-derived growth factor receptor alpha)	imatinib (Gleevec), sorafenib (Nexavar), sunitinib (Sutent), pazopanib (Votrient), nilotinib (Tasigna), axitinib (Inlyta) and dasatinib (Sprycel)
PSMC2 (26S protease regulators subunit 7)	bortezomib (Velcade)
